# Atypical Sensory Processing and Its Correlation with Behavioral Problems in Late Preterm Children at Age Two

**DOI:** 10.3390/ijerph18126438

**Published:** 2021-06-14

**Authors:** Yu-Chin Chen, Wen-Hui Tsai, Chung-Han Ho, Hsuan-Wen Wang, Lan-Wan Wang, Lin-Yu Wang, Hsin-Hua Wang, Yea-Shwu Hwang

**Affiliations:** 1 Department of Pediatrics, Chi Mei Medical Center, Chiali, Tainan 72263, Taiwan; ruby721222@hotmail.com; 2 Division of Neonatology, Department of Pediatrics, Chi Mei Medical Center, Tainan 71004, Taiwan; whys.tsai@gmail.com (W.-H.T.); lanwan@ms67.url.com.tw (L.-W.W.); linyu870203@gmail.com (L.-Y.W.); b101092111@tmu.edu.tw (H.-H.W.); 3 Graduate Institute of Medical Sciences, College of Health Sciences, Chang Jung Christian University, Tainan 711301, Taiwan; 4 Department of Medical Research, Chi Mei Medical Center, Tainan 71004, Taiwan; ho.c.hank@gmail.com; 5 Taipei City Psychiatric Center, Taipei City Hospital, Taipei 11080, Taiwan; wendy19900901@gmail.com; 6 Department of Biotechnology and Food Technology, Southern Taiwan University of Science and Technology, Tainan 71073, Taiwan; 7 Center for General Education, Southern Taiwan University of Science and Technology, Tainan 71073, Taiwan; 8 Department of Pediatrics, Chi Mei Hospital, Liouying, Tainan 73657, Taiwan; 9 Department of Occupational Therapy, College of Medicine, National Cheng Kung University, Tainan 70101, Taiwan

**Keywords:** late preterm, sensory processing, behavioral problems, child

## Abstract

This study aimed to compare the prevalence rate of atypical sensory processing in late preterm (LP) and term children at two years of age and to further investigate the co-occurrence of atypical sensory processing and behavioral problems (internalizing/externalizing) in both groups of children. A total of 104 children (52 LP and 52 sex- and birth order-matched term children) were included. The primary caregivers were asked to complete the Infant/Toddler Sensory Profile—Chinese version and the Child Behavior Checklist 1.5-5Y—Chinese version (CBCL-C/1.5-5). We found that the LP group had a similar prevalence rate of atypical sensory processing to the term group. However, neonatal intensive care unit experience (*r* = −0.356, *p* = 0.013, with visual processing) and days of ventilation and supplementary oxygen (*r* = −0.392, *p* = 0.004, with low registration) after birth were significantly correlated with the atypical sensory processing of LP children. Both LP and term children with behavioral problems seemed to have a higher prevalence rate of atypical sensory processing than their peers without behavioral problems. However, when Bonferroni correction was used to control for the statistical errors of multiple comparisons, only in the LP group did the co-occurrence of atypical sensory processing (auditory and oral sensory processing and sensation avoiding) and behavioral problems reach significance. In conclusion, the influence of late preterm birth on sensory processing may become subtle at age two, with the exception of those LP children experiencing complicated medical management after birth. A high level of co-occurrence of atypical sensory processing and behavioral problems suggests that the administration of a sensory processing assessment may be helpful to clarify the cause of problematic behavior and to recommend an appropriate intervention for LP children with behavioral problems.

## 1. Introduction

The term “late preterm (LP)” refers to those infants born from 34–36 weeks of gestation. This population of infants comprises approximately 70% of preterm births (<37 weeks of gestation at birth) in the United States [1]. The rate of LP births has increased in recent years [2]. Although compared to preterm babies born at less than 34 weeks of gestation, LP infants have a higher survival rate and fewer complications, they still have a higher mortality and more health problems than term infants, defined as birth after 37 weeks of gestation. In the past decade, studies have paid more attention to the developmental outcomes of the LP population and have found that this population is also at an increased risk of having cognitive, motor, and language delays than those infants born at term [3]. However, to date, there is still scant evidence available related to their sensory processing functions, which may impact their development [4,5], emotions and behavior [6,7], daily functioning, and social participation [8]. Given the high prevalence of LP births, it is believed that a greater understanding of their developmental characteristics will be an important matter when developing a proper care model for this population in order to prevent later disabilities.

Sensory processing refers to an individual’s ability to receive, interpret, organize, and react to sensory stimuli (e.g., auditory, tactile, and visual) from their own body and the surrounding environment [9]. Individuals have different responses and behavioral patterns in response to sensory stimuli depending on their neurological thresholds (low/high) and self-regulation strategies (active/passive) [8]. Based on this concept, Dr. Dunn proposed four sensory processing patterns: (1) Persons with a low registration pattern (high threshold/passive strategy) fail to notice sensory stimuli and do not actively look for sensory input; (2) Persons with a sensation seeking pattern (high threshold/active strategy) do not notice sensory stimuli easily but look for sensory input for themselves; (3) Persons with a sensory sensitivity pattern (low threshold/passive strategy) are sensitive and feel distress with sensory stimuli but do not actively avoid them; (4) Persons with a sensation avoiding pattern actively avoid sensory stimuli and control sensory input [8].

It is assumed that the interruption and deprivation of sensory experiences in the womb because of preterm birth substituted with abnormal sensory stimuli (e.g., bright light and noisy, painful medical procedures) when being cared for in the neonatal intensive care unit (NICU) during the perinatal/neonatal period may alter normal brain development, thus negatively impacting the sensory processing abilities in preterm children [10]. This assumption has been supported by studies on early preterm children born below 34 weeks of gestation [11,12,13], but this is yet to be confirmed in those born late preterm due to very limited evidence [14].

Previous studies on preterm children born before 34 weeks of gestation have consistently indicated that a high percentage (37–82%) of them exhibit atypical sensory processing [5,11,13,15]. Such atypical sensory processing is continuously present from infancy into childhood [12]. However, few studies have explored the sensory processing functions of LP children. One study by Bart et al. compared the sensory processing of LP (34–35 weeks of gestation) and term (≥37 weeks of gestation) infants at one year of age using two assessments—a performance-based tool (the Test of Sensory Function in Infants (TSFI)) and a caregiver-reported questionnaire (the Infant/Toddler Sensory Profile (ITSP)) [14]. Similar to the findings on very and extremely preterm babies, they also found that a higher percentage of LP infants showed atypical sensory processing compared to the term controls. More LP infants exhibited at-risk and deficient performance in all subscales (i.e., deep pressure, adaptive motor, visual-tactile integration, and vestibular reaction) of the TSFI. However, on the ITSP, the only significant differences between groups were in the auditory (26.2% vs. 7.4%) and oral sensory (33% vs. 11.1%) functions [14]. Sensory processing functioning beyond 12 months of age in this population is thus still not clear.

Sensory processing is known to affect a child’s developmental and behavioral performance [16]. In the preterm population, previous studies have found that sensory processing is related to development (e.g., motor, cognitive, and language) [5,13], executive function [15], and feeding performance [17]. However, little is known about the relationship between sensory processing and behavioral problems in this population. A significant correlation between atypical sensory processing and behavioral problems has been demonstrated in general [18] and clinical child populations (e.g., autism spectrum disorders and fetal alcohol spectrum disorders) [6,7,19]. A few studies have further indicated there are some atypical sensory processing behaviors particularly correlated with behavioral problems. For example, Tseng et al. found that sensation avoiding was the most important predictor and explained 48% of the variance of internalizing problems (e.g., anxious/depressed, somatic complaints, and withdrawn) for four- to seven-year-old children with typical development. For externalizing behaviors (e.g., inattention, aggression), sensation seeking was the specific predictor and explained 26% of the variance of this problematic behavior [7]. Based on these findings, it is assumed that atypical or dysfunctional sensory processing may lead to behavioral problems in a child.

In addition to increased developmental risks, some evidence has shown that a greater percentage of school-aged children born late preterm exhibit behavioral difficulties (e.g., internalizing behavioral problems and hyperactive/inattention) than their peers born at term [20,21]. However, the underlying causes for their behavioral problems are uncertain. Child behavioral problems often cause stress in parents and poor child–parent relationships [19]. Identifying the underlying causes of such behavioral problems is very important, since it can not only lead to an effective intervention intended to improve the behavioral performance in these children, but also may relieve parental stress and improve wellbeing. Whether atypical sensory processing in LP children contributes to their behavioral problems requires further investigation.

To date, only one study has found that atypical sensory processing is more prevalent in LP infants than in their term peers at one year of age [14]. Little is known about the sensory processing of LP children beyond infancy. Furthermore, the relationship between atypical sensory processing and behavioral problems in this population is unclear. Therefore, there were two aims in the present study: (1) to investigate the sensory processing patterns of LP children at two years of age in comparison to term born peers, and (2) to compare the prevalence rates of atypical sensory processing in children with and without behavioral problems (i.e., internalizing and externalizing behaviors) in both LP and term groups.

## 2. Materials and Methods

### 2.1. Participants

The participants included two-year-old LP and term children. All candidate participants were born at a medical center in Tainan, Taiwan, and recruited from October 2013 to June 2016. A flyer was mailed to the parents of the candidate participants that invited them to participate in the study. Parents who wanted to participate in this study were asked to contact the research assistant by phone, and further screening was conducted at that time. The inclusive criteria for the preterm group included children born at 34^0/7^ through 36^6/7^ weeks of gestational age to native Chinese-speaking parents. Preterm children who had (1) congenital anomalies, (2) genetic or chromosome abnormalities, (3) auditory or visual problems that could not be corrected to be normal, (4) a history of brain insults (e.g., intraventribular hemorrhage or asphyxia), or (5) being cared for in a day center were excluded.

The inclusive criteria for the term group were (1) ≥37 weeks and <42 weeks of gestation at birth, (2) birth weight ≥2500 g, (3) no health problems at birth, and (4) having native Chinese-speaking parents. The exclusion criteria for term children were the same as those for the LP group. Considering the confounding effects of sex and birth order on the results of sensory processing and behavioral assessments [22,23], term children who were sex- and first born-matched to the preterm group were selected as the control group.

This study was approved by the Institutional Review Board of Chi Mei Medical Center, Tainan, Taiwan (10208-013). Written informed consent was obtained from all parents before data collection. 

### 2.2. Measures

#### 2.2.1. Infant/Toddler Sensory Profile (ITSP)

The ITSP was developed by Dr. Dunn [24] to assess sensory processing in 0–36-month-old children. There are two versions included in the ITSP, one with 36 items for infants 0–6 months of age and the other with 48 items for infants 7–36 months of age. The questions in the ITSP are rated by the primary caregiver using a five-point Likert scale (always, often, occasionally, rarely, and never) based on the frequency of their child’s responses to various types of sensory stimuli in daily life [24]. The ITSP generates two types of raw scores, namely section and quadrant scores. The former includes (1) auditory, (2) visual, (3) tactile, (4) vestibular, and (5) oral sensory processing scores. The latter includes (1) low registration, (2) sensation seeking, (3) sensory sensitivity, and (4) sensation avoidance. Lower scores represent a higher frequency of section (quadrant) behavior. Scores below −1 standard deviations (SD) from the mean of the norm represent atypically frequent performance (i.e., probably and definitely different) [24].

A Chinese version of the ITSP (ITSP-C) with a norm of Taiwanese infants and young children aged at 0–36 months has been established [25]. Both the ITSP [24] and the ITSP-C [23,25] have sound levels of reliability and validity.

#### 2.2.2. Child Behavior Checklist 1.5–5Y (CBCL/1.5–5)

This caregiver-reported scale is used to evaluate emotional and behavioral problems in children aged 1.5–5 years old. There are 100 questions in total, with 99 questions assessing specific behavioral problems and a blank question [26]. Caregivers use a three-point Likert scale to score whether the description of each item is true for their child’s behavior in the past two months. The questions on the CBCL/1.5–5 can be classified into seven narrow-band behavioral syndromes (emotionally reactive, anxious/depressed, somatic complaints, withdrawn, sleep problems, attentional problems, and aggressive behavior) and other problems (not classified), which comprise the total problems scale. The questions in four narrow-band syndromes (emotionally reactive, anxious/depressed, somatic complaints, and withdrawn) further constitute the internalizing scale, and two other syndromes (attentional problems and aggressive behavior) constitute the externalizing scale [26]. A higher score indicates a greater severity of symptoms.

A Chinese version of the CBCL/1.5–5 (CBCL-C/1.5–5) has been established but lacks Taiwanese children’s norms at the present time [27]. Therefore, the participants’ raw scores were converted into T scores based on the norm of American children at 1.5–5 years of age; a T score for the internalizing, externalizing, and total problems scales of 64 or greater reflects the child’s symptoms to be in the clinical range [27]. The CBCL-C/1.5–5 has good to excellent levels of reliability (internal consistency, test–retest reliability, and inter-parent agreement) and acceptable construct validity [22].

### 2.3. Procedure

Children who met the criteria were invited to come to our department for a cognitive and motor development evaluation using the Bayley Scales of Infant and Toddler Development-III (Bayley-III, PsychCorp: San Antonio, TX, USA) [28] scheduled within two weeks after they were chronologically two years of age (term children) or corrected at two years of age (LP children). At the same time, the mothers were asked to fill out the ITSP-C, the CBCL-C/1.5–5, and the child demographic questionnaire.

### 2.4. Data Analysis

Demographic and neonatal data and developmental scores on the Bayley III for the LP and term groups were compared using independent *t*-tests for the continuous variables and chi-square tests for the categorical variables. The results of the Kolmogorov–Smirnov tests showed that some of the ITSP-C raw scores did not exhibit a normal distribution. Thus, Mann–Whitney *U*-tests were used to analyze the differences between the LP and term groups on the ITSP-C scores. The percentage of children with atypical sensory processing patterns (raw score < norm mean −1 SD) in the LP and term groups were compared using chi-square or Fisher’s exact tests depending on the number of children in each group. Spearman’s correlation coefficients were used to determine the association between each demographic and neonatal variable and the sensory section and quadrant scores. To investigate the prevalence of the co-occurrence of atypical sensory processing and behavioral problems (internalizing or externalizing T score at 64 or greater) in the LP and term groups, the number of children with atypical sensory processing in the two behavioral subgroups (with and without behavioral problems) was compared using the Fischer’s exact tests. All data were analyzed using SPSS 17.0 software (SPSS Inc., Chicago, IL, USA). A *p* value of <0.0056 was considered statistically significant when Bonferroni correction was used to control the statistic errors of multiple comparisons in the analyses.

## 3. Results

### 3.1. Child Characteristics

A total of 52 LP and 52 sex- and first born-matched children born at term were included. The sample characteristics are shown in Table 1. A lower gestational week (*p* < 0.001) and weight (*p* < 0.001) at birth and more complicated neonatal medical events were found in the LP group. There were no between-group differences in the socioeconomical status scores and maternal education. Three children (two LP children and one term child) had a diagnosis of developmental delay and all of them received early intervention at the time of the study. Cognitive and motor scores on the Bayley III did not differ between the two groups (Table 1).

### 3.2. Results of the ITSP-C

The sensory processing abilities of the LP and term children are described in Table 2. A similar raw score in each section and quadrant was shown in the LP and term children. There were also no significant different in the percentage of children with atypical sensory section and quadrant scores in both groups (Table 2). Although a greater percentage of children in the LP group exhibited atypical auditory processing than those born at term (25% vs. 10%, *p* = 0.038), the difference did not reach statistical significance when Bonferroni correction was conducted (*p* < 0.0056).

In the LP group, the Spearman correlation results revealed a significant correlation between experience of staying in the NICU and the visual section score (*r* = −0.356, *p* = 0.013), as well as days of ventilation and supplementary oxygen and low registration scores (*r* = −0.392, *p* = 0.004), indicating that both neonatal factors may be risk factors for atypical sensory processing functions. A significant correlation between sex and vestibular section score (*r* = 0.343, *p* = 0.013) was found, indicating that the boys had more frequent vestibular processing behaviors. The other demographic and neonatal factors did not correlate with any section or quadrant scores.

### 3.3. Relationship between Atypical Sensory Processing and Behavioral Problems in LP Children

The prevalence rates of atypical sensory processing in the LP children with and without behavioral problems are shown in Table 3. When Bonferroni correction was conducted (*p* < 0.0056), a higher prevalence rate of atypical sensory processing was still shown in the LP children with behavioral problems. A greater percentage of LP children with internalizing problems showed atypical auditory (*p* = 0.001) and oral sensory processing (*p* = 0.004), as well as sensation avoiding (*p* < 0.001), than those without internalizing problems. Similarly, a significantly greater LP children with externalizing symptoms had atypical auditory processing (*p* = 0.002) and sensation avoiding patterns (*p* = 0.002). 

### 3.4. Relationship between Atypical Sensory Processing and Behavioral Problems in Term Children

The prevalence rates of atypical sensory processing in the term children with and without behavioral problems are shown in Table 4. Although more children with internalizing behaviors showed a sensation avoiding pattern than those without behavioral problems (42% vs. 7%), the group difference did not reach statistical significance when the *p*-value was set at 0.0056 because of Bonferroni correction. Similarly, atypical visual processing (40% vs. 4%) and sensation avoiding (60% vs. 11%) were more commonly seen in those term children with externalizing problems than those without problems, but the differences were also non-significant.

## 4. Discussion

To date, few studies have investigated sensory processing in LP children. We found only one study addressing this function in LP infants at 12 months of age [14]. Therefore, the present results contribute to the body of knowledge on the development of the sensory processing function in this population beyond 12 months of age and its associations with their behavioral problems.

In the present study, we found that LP children had comparable sensory processing performance with the term-born controls in all sections and quadrants of the ITSP at two years of age. In Bart et al.’s study on 103 one-year-old LP and 27 term infants, they found a greater percentage of atypical auditory processing in the LP group (26.2% vs. 7.4%) with a *p*-value of 0.039 [14]. Our LP and term samples had similar prevalence rates (25% vs. 10%) and *p*-value (0.038) to theirs. However, in the present study, the group difference in auditory processing was no more significant when Bonferroni *p* correction was made for multiple comparisons. The results of both small studies indicated that there may be a trend for more LP children to have atypical auditory processing than term controls based on the behavioral observation of caregivers. Future research using neurophysiological assessments such as auditory event-related potentials [31] may provide direct, objective evidence of the brain processing activity in terms of the differences between LP and term children’s responses to auditory stimuli.

Different from Bart et al.’s findings on the ITSP, we did not find a higher prevalence of atypical oral sensory processing behavior in our two-year-old LP children compared to the controls. One possible explanation for this inconsistency is that the atypical oral sensory processing performance found in LP infants may be a transient phenomenon, since neural maturation, repeated exposure to various oral sensory stimuli (e.g., eating different kinds of textured food, mouthing hands, toys, and objects), and gradual habituation or desensitization to oral stimuli [32] beyond one year old may lessen atypical oral sensory processing behavior in LP children.

In the case of neonatal factors, we found that NICU stay and longer days of ventilation and supplementary oxygen were correlated with stronger sensory processing behavior (i.e., low registration and visual processing) in the LP children. Our findings are consistent with those of a study on preschoolers born ≤32 weeks of gestation, which also revealed that those with atypical sensory processing patterns had a longer stay in the NICU and more days of ventilation [11]. These results suggest that preterm children requiring complicated medical care after birth may be at higher risk of having atypical sensory processing, perhaps because they experience more stressful events (e.g., more severity of complications and suction) and aversive sensory stimuli (e.g., bright light and noise in the NICU), which may in turn interfere with normal brain development [10]. The sensory processing function of those LP children with a complicated medical history at birth needs further investigation. On the contrary, in contrast to Bart et al.’s findings, we did not find a correlation between the gestational age and sensory processing scores in our sample. One reason for this difference may have been the low number of children born at 34 weeks of gestation (*n* = 7) in the present sample and thus the absence of enough statistical power to show an association. In addition, a low correlation (*r* = 0.343) of sex and vestibular section scores in the LP group found in this study showed that LP boys tended to have a stronger vestibular processing pattern than LP girls. This may be due to a higher percentage of LP boys enjoying vestibular stimulation activities reported by their caregivers on the ITSP (i.e., “My child enjoys physical activity, like bouncing and being held up high in the air” and “My child enjoys rhythmical activities, like swing, rocking, and car rides”).

In the present study, LP children with behavioral problems were more likely to exhibit atypical sensory processing. However, we failed to find a significant co-occurrence of atypical sensory processing and behavioral problems in the term group. Caution should be taken when interpreting the present results. Particularly for the term children, the absence of a significant finding between atypical sensory processing and behavioral problems may perhaps be due to a very limited number of term children with behavioral problems (*n* = 12 with internalizing problems and *n* = 5 with externalizing problems); thus, we had insufficient power to show any statistical differences. A larger study is required to verify the relationships between atypical sensory processing and behavioral problems for term children.

Because evidence about the relationship between sensory processing and behavioral problems in the preterm population or in children below three years of age is scarce, the present findings on LP children had no proper child population for comparison. However, in the line of previous findings on preschoolers and school-aged general and clinical children [6,7,18,19], our results based on two-year-old children born late preterm also support a high level of co-occurrence of atypical sensory processing and behavioral problems. These results suggest that sensory processing-based behavioral problems may exist in quite a few children, including young children born at late preterm. Therefore, we suggest that a direct or parent-reported sensory processing evaluation could be administered on LP children with behavioral problems. This may help clarify the cause for their problematic behaviors and provide an appropriate intervention for them.

On the contrary, of four sensory quadrant patterns on the sensory profile (i.e., low registration, sensation seeking, sensory sensitivity, and sensation avoiding), which one specifically correlated with child’s internalizing or externalizing problems is still controversial across the studies [7,18]. These inconsistent findings may be attributed to a variety of populations (e.g., age, diagnosis, and races) and methodological differences (e.g., behavioral assessment tools and statistical methods used for data analysis). In the present study, a high level of coexistence of sensation avoiding with internalizing (50%) and externalizing (56%) problems was noted in the LP children. Based on Dunn’s model of sensory processing, children with sensation avoiding means more easily noticing environmental stimuli and often use active self-regulation strategies (e.g., withdrawing or not involving themselves) to avoid challenging (e.g., over-stimulating, new, or unfamiliar) activities or environments [8]. For those LP children with a sensation avoiding pattern, some strategies, such as decreasing the amount of new sensory input within an activity or environment, particularly the sensations the child tends to avoid, as well as creating routines for daily tasks, may be helpful to improve their behavioral performance in daily life [8]. In addition, LP children with internalizing problems tended to exhibit atypical auditory and oral sensory processing. Compared to peers without internalizing problems, more of them showed sensitive to auditory (i.e., distracted and/or difficulty eating in noisy environment) and oral stimuli (i.e., resist having teeth brushed), but on the contrary liked to seek oral stimuli (i.e., lick/chew nonfood objects or mouth objects). For LP children with externalizing problems, they also tended to be sensitive to auditory stimuli (i.e., startle easily at sound, compared to other children the same age).

A few limitations exist in the present study. First, the present LP and term samples were collected from a single medical center. Furthermore, the limited statistical power due to a small number in some subgroups may have decreased the chance to detect differences between the groups. Therefore, caution should be taken when generating the present findings to other two-year-old children born late preterm and term, and larger sample sizes are required in the future research. Second, five item questions scattered in the sensory sections (quadrants) of the ITSP are similar to seven item questions in the CBCL. They may potentially have a chance to contribute to the co-occurrence between atypical sensory processing and behavioral problems. Third, the present findings rely on the same caregiver’s report of their child’s sensory processing and behavioral difficulties, which may have potentially biased the relationship between sensory processing and behavioral problems. If possible, asking a separate caregiver (e.g., father or nanny) to fill out one of the two questionnaires may prevent intra-rater bias. 

## 5. Conclusions

The present evidence indicates that the sensory processing patterns in LP and term children at two years of age are comparable in terms of the behavioral observations and reports of caregivers on the ITSP. Future studies using neurophysiological assessment tools are required to provide more direct evidence regarding the sensory processing of LP children. The significant correlation between neonatal risk factors and sensory processing found in this study revealed that LP children with complicated medical experiences after birth may be at higher risk of atypical sensory processing patterns. In addition, we found a high level of co-occurrence of atypical sensory processing (i.e., auditory and oral sensory processing and sensation avoiding) and internalizing/externalizing problems in LP children. This may infer that sensory processing-based behavioral problems exist in these LP children with behavioral problems. Therefore, it is suggested that the administration of a sensory processing assessment and strategies is required for LP children with behavioral problems.

## Figures and Tables

**Table 1:** The children’s characteristics.

Variable	Mean (SD)	
	Late Preterm (*n* = 52)	Term (*n* = 52)
Age (months) ^a^	24.3 (0.2)	24.3 (0.2)
Male, *n* (%)	29 (55.8)	29 (55.8)
Neonatal medical history		
Gestational age (weeks)	35.7 (0.8)	39.0 (1.0) **
Birth weight (g)	2283 (429)	3200 (286) **
Head circumference (cm)	32.4 (2.6)	34.1 (1.2)
SGA, *n* (%)	15 (28.8)	0 (0)
Ventilation need (yes), *n* (%)	8 (15.4)	0 (0)
Days in ventilator and oxygen	2.4 (2.6)	0 (0)
NICU stay (yes), *n* (%)	9 (17.3)	0 (0)
Hospital stay (days)	10.0 (8.4)	3.9 (1.2)
Family socioeconomical score ^a^	37.5 (11.6)	38.7 (8.7)
Maternal education (≥university), *n* (%)	34 (65.4)	37 (72.5)
First-born child, *n* (%)	19 (36.5)	19 (36.5)
No. of children with a diagnosis of developmental delays	2 (1 motor, 1 language)	1 (motor)
Bayley-III composite score ^b^		
Cognition	107.0 (13.9)	103.6 (8.0)
Motor	109.0 (11.9)	105.0 (11.9)

SGA, small for gestational age; NICU, neonatal intensive care unit. Bayley III, Bayley Scale of Infant and Toddler Development—3rd version. ^a^ Calculated using the levels of maternal education and occupation. Scores classified into I (very high) to V (very low): I = 52–55, II = 41–51, III = 30–40, IV = 19–29, and V = 11–18 [29]. ^b^ Corrected age used for late preterm children. The norm mean is 100 (SD = 15) and a composite score <85 (norm mean −1 SD) defined as developmental delay [30]. * *p* < 0.05, ** *p* < 0.01.

**Table 2:** Performance on the ITSP-C in the late preterm and term groups.

ITSP-C	Score, Mean (SD)			Atypical Performance ^a^, *n* (%)		
	Late Preterm (*n* = 52)	Term (*n* = 52)	*p*	Late Preterm (*n* = 52)	Term (*n* = 52)	*p*
Section						
Auditory	36.5 (5.2)	36.2 (4.5)	0.400	13 (25)	5 (10)	0.038
Visual	21.9 (3.0)	21.5 (2.7)	0.534	4 (8)	4 (8)	1.000
Tactile	50.7 (6.7)	51.3 (7.0)	0.750	8 (15)	9 (17)	0.791
Vestibular	20.4 (3.3)	20.0 (2.4)	0.569	3 (6)	3 (6)	1.000
Oral sensory	27.3 (4.5)	26.4 (4.0)	0.213	6 (12)	6 (12)	1.000
Quadrant						
Low registration	47.8 (4.8)	47.2 (4.3)	0.419	6 (12)	6 (12)	1.000
Sensation seeking	31.8 (7.8)	31.9 (6.4)	0.868	4 (8)	3 (6)	1.000
Sensory sensitivity	43.8 (5.9)	43.0 (5.5)	0.883	5 (10)	2 (4)	0.437
Sensation avoiding	45.3 (6.3)	44.8 (5.9)	0.600	8 (15)	8 (15)	1.000

ITSP-C, Infant/Toddler Sensory Profile—Chinese version; ^a^ The number of children with a raw score < −1 SD from the norm mean for the ITSP-C. * *p* < 0.0056 due to Bonferroni correction.

**Table 3:** The prevalence of atypical ITSP-C performance in late preterm children with and without behavioral problems.

Atypical ITSP-C Performance	CBCL-C, *n* (%)					
	Internalizing			Externalizing		
	Typical (*n* = 37)	>Clinical ^a^ (*n* = 14)	*p*	Typical (*n* = 42)	Clinical ^a^ (*n* = 9)	*p*
Section						
Auditory	5 (14)	8 (57)	0.001 *	7 (17)	6 (67)	0.002 *
Visual	2 (5)	2 (14)	0.300	3 (7)	1 (11)	0.552
Tactile	4 (11)	4 (29)	0.192	5 (12)	3 (33)	0.137
Vestibular	0 (0)	3 (21)	0.017	1 (2)	2 (22)	0.077
Oral sensory	1 (3)	5 (36)	0.004 *	3 (7)	3 (33)	0.060
Quadrant						
Low registration	4 (11)	2 (14)	0.661	5 (12)	1 (11)	1.000
Sensation seeking	1 (3)	3 (21)	0.058	2 (5)	2 (22)	0.139
Sensory sensitivity	2 (5)	3 (21)	0.120	4 (10)	1 (11)	1.000
Sensation avoiding	1 (3)	7 (50)	<0.001 *	3 (7)	5 (56)	0.002 *

ITSP-C, Infant/Toddler Sensory Profile—Chinese version; CBCL-C, Child Behavior Checklist—Chinese version. ^a^ A T score of 64 or greater (>90% of the norm); Fisher’s exact tests used. * *p* < 0.0056 due to Bonferroni correction.

**Table 4:** The prevalence of atypical ITSP-C performance in term children with and without behavioral problems.

Atypical ITSP-C	CBCL-C, *n* (%)					
	Internalizing			Externalizing		
	Typical (*n* = 39)	Clinical ^a^ (*n* = 12)	*p*	Typical (*n* = 46)	Clinical ^a^ (*n* = 5)	*p*
Section						
Auditory	2 (5)	3 (25)	0.078	3 (7)	2 (40)	0.069
Visual	3 (7)	1 (8)	1.000	2 (4)	2 (40)	0.043
Tactile	6 (15)	3 (25)	0.424	8 (17)	1 (20)	1.000
Vestibular	2 (5)	1 (8)	0.561	2 (4)	1 (20)	0.271
Oral sensory	4 (10)	2 (17)	0.616	4 (9)	2 (40)	0.099
Quadrant						
Low registration	4 (10)	2 (17)	0.616	4 (9)	2 (40)	0.099
Sensation seeking	3 (7)	0 (0)	1.000	3 (7)	0 (0)	1.000
Sensory sensitivity	0 (0)	2 (17)	0.052	1 (2)	1 (20)	0.188
Sensation avoiding	3 (7)	5 (42)	0.012	5 (11)	3 (60)	0.023

ITSP-C, Infant/Toddler Sensory Profile—Chinese version; CBCL-C, Child Behavior Checklist—Chinese version. ^a^ A T score of 64 or greater (>90% of the norm); Fisher’s exact tests used. * *p* < 0.0056 due to Bonferroni correction.

## Data Availability

The dataset used and analyzed during the current study is available from the corresponding author upon reasonable request.

## References

[1] Martin J.A., Hamilton B.E., Osterman M.J.K. (2019). Births in the United States, 2018.

[2] Stewart D.L., Barfield W.D. (2019). Committee on Fetus and Newborn Updates on an At-Risk Population: Late-Preterm and Early-Term Infants. Pediatrics.

[3] Woythaler M. (2019). Neurodevelopmental outcomes of the late preterm infant. Semin. Fetal Neonatal Med..

[4] Celik H.I., Elbasan B., Gucuyener K., Kayihan H., Huri M. (2018). Investigation of the Relationship between Sensory Processing and Motor Development in Preterm Infants. Am. J. Occup. Ther..

[5] Eeles A.L., Anderson P.J., Brown N.C., Lee K.J., Boyd R.N., Spittle A.J., Doyle L.W. (2013). Sensory profiles obtained from parental reports correlate with independent assessments of development in very preterm children at 2 years of age. Early Hum. Dev..

[6] Franklin L., Deitz J., Jirikowic T., Astley S. (2008). Children with fetal alcohol spectrum disorders: Problem behaviors and sensory processing. Am. J. Occup. Ther..

[7] Tseng M.-H., Fu C.-P., Cermak S.A., Lu L., Shieh J.-Y. (2011). Emotional and behavioral problems in preschool children with autism: Relationship with sensory processing dysfunction. Res. Autism Spectr. Disord..

[8] Dunn W. (2007). Supporting Children to Participate Successfully in Everyday Life by Using Sensory Processing Knowledge. Infants Young Child..

[9] Miller L.J., Anzalone M.E., Lane S.J., Cermak S.A., Osten E.T. (2007). Concept Evolution in Sensory Integration: A Proposed Nosology for Diagnosis. Am. J. Occup. Ther..

[10] El-Metwally D.E., Medina A.E. (2020). The potential effects of NICU environment and multisensory stimulation in prematurity. Pediatr. Res..

[11] Crozier S.C., Goodson J.Z., Mackay M.L., Synnes A.R., Grunau R.E., Miller S.P., Zwicker J.G. (2016). Sensory Processing Patterns in Children Born Very Preterm. Am. J. Occup. Ther..

[12] Wickremasinghe A.C., E Rogers E., Johnson B.C., Shen A., Barkovich A.J., Marco E.J. (2013). Children born prematurely have atypical Sensory Profiles. J. Perinatol..

[13] Chorna O., E Solomon J., Slaughter J.C., Stark A.R., Maitre N.L. (2014). Abnormal sensory reactivity in preterm infants during the first year correlates with adverse neurodevelopmental outcomes at 2 years of age. Arch. Dis. Child. Fetal Neonatal Ed..

[14] Bart O., Shayevits S., Gabis L., Morag I. (2011). Prediction of participation and sensory modulation of late preterm infants at 12 months: A prospective study. Res. Dev. Disabil..

[15] Adams J.N., Feldman H.M., Huffman L.C., Loe I.M. (2015). Sensory processing in preterm preschoolers and its association with executive function. Early Hum. Dev..

[16] Miller L.J., Fuller D.A., Roetenberg J. (2014). Sensational Kids: Hope and Help for Children with Sensory Processing Disorder (SPD).

[17] Kim M.-S., Kim K.-M., Chang M.-Y., Hong E. (2019). A Study on Correlation of Sensory Processing ability With Feeding of Preterm Infants and Toddlers. J. Korean Soc. Sens. Integr. Ther..

[18] Dean E.E., Little L., Tomchek S., Dunn W. (2018). Sensory Processing in the General Population: Adaptability, Resiliency, and Challenging Behavior. Am. J. Occup. Ther..

[19] Gourley L., Wind C., Henninger E.M., Chinitz S. (2012). Sensory Processing Difficulties, Behavioral Problems, and Parental Stress in a Clinical Population of Young Children. J. Child Fam. Stud..

[20] Brumbaugh J.E., Conrad A.L., Lee J.K., Devolder I.J., Zimmerman M.B., Magnotta V., Axelson E.D., Nopoulos P.C. (2016). Altered brain function, structure, and developmental trajectory in children born late preterm. Pediatr. Res..

[21] Polic B., Bubić A., Mestrovic J., Markić J., Kovacevic T., Furlan I.A., Utrobicić I., Kolčić I. (2017). Emotional and behavioral outcomes and quality of life in school-age children born as late preterm: Retrospective cohort study. Croat. Med. J..

[22] Wu Y.-T., Chen W.J., Hsieh W.-S., Chen P.-C., Liao H.-F., Su Y.-N., Jeng S.-F. (2012). Maternal-reported behavioral and emotional problems in Taiwanese preschool children. Res. Dev. Disabil..

[23] Yang C.-Y., Tseng M.-H., Cermak S.A., Lu L., Shieh J.-Y. (2020). Reliability and Validity of the Chinese Version of the Infant/Toddler Sensory Profile. Am. J. Occup. Ther..

[24] Dunn W., Daniels D.B. (2002). Initial development of the infant/toddler sensory profile. J. Early Interv..

[25] Tseng M.H., Yang C.Y. (2009). Infant/Toddlers Sensory Profile-Chinese Version.

[26] Achenbach T.M., Rescorla L.A. (2000). Manual for the ASEBA Preschool Forms and Profiles.

[27] Chen Y., Huang H.L., Chao C.C. (2009). Achenbach System of Empirically Based Assessment (Chinese Version).

[28] Bayley N. (2006). Bayley Scales of Infant and Toddler Development: Administration Manual.

[29] Lin S.C. (2002). Educational Sociology.

[30] Johnson S., Moore T., Marlow N. (2014). Using the Bayley-III to assess neurodevelopmental delay: Which cut-off should be used?. Pediatr. Res..

[31] Stene-Larsen K., Brandlistuen R.E., Lang A.M., Landolt M.A., Latal B., Vollrath M.E. (2014). Communication Impairments in Early Term and Late Preterm Children: A Prospective Cohort Study following Children to Age 36 Months. J. Pediatr..

[32] Case-Smith J., O’Brien J.C. (2015). Occupational Therapy for Children and Adolescents.

